# Short-term repeated corticosterone administration enhances glutamatergic but not GABAergic transmission in the rat motor cortex

**DOI:** 10.1007/s00424-015-1773-6

**Published:** 2015-12-23

**Authors:** Joanna Kula, Anna Blasiak, Anna Czerw, Grzegorz Tylko, Joanna Sowa, Grzegorz Hess

**Affiliations:** Institute of Zoology, Jagiellonian University, Gronostajowa 9, 30-387 Krakow, Poland; Department of Physiology, Institute of Pharmacology, Polish Academy of Sciences, Smetna 12, 31-343 Krakow, Poland

**Keywords:** Brain slices, Model of stress, Neocortex

## Abstract

It has been demonstrated that stress impairs performance of skilled reaching and walking tasks in rats due to the action of glucocorticoids involved in the stress response. Skilled reaching and walking are controlled by the primary motor cortex (M1); however, it is not known whether stress-related impairments in skilled motor tasks are related to functional and/or structural alterations within the M1. We studied the effects of single and repeated injections of corticosterone (twice daily for 7 days) on spontaneous excitatory and inhibitory postsynaptic currents (sEPSCs and sIPSCs) recorded from layer II/III pyramidal neurons in ex vivo slices of the M1, prepared 2 days after the last administration of the hormone. We also measured the density of dendritic spines on pyramidal cells and the protein levels of selected subunits of AMPA, NMDA, and GABA_A_ receptors after repeated corticosterone administration. Repeatedly administered corticosterone induced an increase in the frequency but not in the amplitude of sEPSCs, while a single administration had no effect on the recorded excitatory currents. The frequency and amplitude of sIPSCs as well as the excitability of pyramidal cells were changed neither after single nor after repeated corticosterone administration. Treatment with corticosterone for 7 days did not modify the density of dendritic spines on pyramidal neurons. Corticosterone influenced neither the protein levels of GluA1, GluA2, GluN1, GluN2A, and GluN2B subunits of glutamate receptors nor those of α_1_, β_2_, and γ_2_ subunits of the GABA_A_ receptor. The increase in sEPSCs frequency induced by repeated corticosterone administration faded out within 7 days. These data indicate that prolonged administration of exogenous corticosterone selectively and reversibly enhances glutamatergic, but not GABAergic transmission in the rat motor cortex. Our results suggest that corticosterone treatment results in an enhancement of spontaneous glutamate release from presynaptic terminals in the M1 and thereby uncovers a potential mechanism underlying stress-induced motor functions impairment.

## Introduction

In the rat brain, the primary motor cortex (M1) contains motor maps, which are representations of how movements are organized [8, 32]. Examples of M1-controlled motor functions in the rat include skilled limb use in reaching for food [49] and in rung ladder walking [1]. Motor skill learning is associated with a reorganization of movement representations within the rat M1 [19, 36]. The mechanism of motor skill learning-related plasticity of the M1 involves activity-dependent, long-term strengthening of excitatory synaptic connections within the cortical layer II/III [37–39]. Motor skill learning has also been reported to induce an increase in synapse number in the M1 [20]. It has been shown that acute and chronic stress impairs the movement accuracy in skilled reaching and walking tasks and alters skilled movement patterns in the reaching task [21, 29]. Stress-related impairments in skilled motor tasks may be related to functional and/or structural plasticity of excitatory synapses within rat M1; however, this possibility has not yet been investigated.

While the effects of acute stress on the brain are usually transient, chronic stress is thought to exert a persistent, detrimental influence, which is generally attributed to a prolonged hyperactivation of the hypothalamic–pituitary–adrenal (HPA) axis and an elevated level of corticosteroids acting at the cellular level through glucocorticoid and mineralocorticoid receptors (GRs and MRs, respectively; reviewed in [5, 25, 33]). Repeated stress has been reported to induce suppression of glutamatergic transmission and a decrease in the expression level of glutamate receptors and synaptic proteins in the rat medial prefrontal cortex (mPFC), which is an important target for glucocorticoids involved in the stress response [24, 51]. Moreover, repeated stress results in profound morphological changes of pyramidal neurons in the mPFC (reviewed in [41]). GR and MR receptors are expressed in the rat M1 [30, 40], and it has been reported that MR and GR blockade ameliorates motor impairments resulting from stress [15]. However, the effects of stress on the structure and function of the rat motor cortex remain largely unknown.

Since 21 days of repeated corticosterone injections reliably increase depression-like behavior [11], repeated corticosterone administration has been proposed as a preclinical rat model of chronic stress (reviewed in [42]). One advantage of this model is that it directly examines the influence of an elevated level of corticosterone on the organism in contrast to different paradigms of repeated behavioral or social stress. Similar to repeated restraint stress, repeated administration of exogenous corticosterone impairs the movement accuracy in skilled reaching and walking tasks [21, 29]. We have previously investigated the effects of repeated corticosterone administration lasting 7 and 21 days, on field potentials evoked in layer II/III of the rat motor cortex [4]. We have reported that corticosterone treatment resulted in an increase in the field potential amplitude which occurred within less than 7 days of corticosterone administration and persisted during the following 2 weeks of the treatment. This effect was accompanied by an increase in the mean frequency of spontaneous excitatory postsynaptic currents (sEPSCs) in layer II/III pyramidal neurons, collectively suggesting an enhancement of excitatory synaptic transmission due to corticosterone treatment.

The aim of the present study was to determine whether the repeated corticosterone administration-induced functional changes in the M1 excitatory synaptic transmission are associated with structural modifications. To this end, we measured the density of dendritic spines in layer II/III pyramidal neurons in control and corticosterone-treated rats. Since stress has previously been reported to induce an increase in the level of GABAergic markers in rat mPFC [9], we also aimed at finding if corticosterone treatment lasting 7 days influences spontaneous inhibitory postsynaptic currents (sIPSCs) in M1 layer II/III pyramidal cells. Moreover, we have also investigated the effects of a single administration of corticosterone on sEPSCs and sIPSCs and assessed the influence of prolonged corticosterone treatment on the protein level of selected subunits of AMPA, NMDA, and GABA_A_ receptors.

## Experimental procedures

### Animals and treatment

Experimental procedures were approved by the Animal Care and Use Committee at the Jagiellonian University and were carried out in accordance with the European Community guidelines for the use of experimental animals and the national law. Male Wistar rats, aged 5–6 weeks at the beginning of the experiment, were housed in groups and maintained on a 12-h light/dark schedule (light on: 0800–2000 hours). Standard food and tap water were available ad libitum. Animals were weighed on a daily basis. Corticosterone (Sigma-Aldrich), suspended in 1 % Tween 80, was administered subcutaneously (dose 10 mg/kg, volume 2 ml/kg) either once (termed: 1× or single administration) or twice daily, for 7 days (termed 7d or repeated administration [52]). Control animals received the vehicle (termed Tw or Tween), but otherwise they were handled identically and were investigated concurrently with corticosterone-treated rats.

### Tissue preparation

In most experiments, brain slices were prepared 2 days after the last corticosterone administration to avoid acute effects of the hormone. In two sets of experiments, slices were prepared 4 or 7 days after the end of corticosterone treatment. Rats were anesthetized with isoflurane (Aerrane, Baxter) and decapitated. Their brains were quickly removed and placed in ice-cold artificial cerebrospinal fluid (ACSF) containing (in mM): 130 NaCl, 5 KCl, 2.5 CaCl_2_, 1.3 MgSO_4_, 1.25 KH_2_PO_4_, 26 NaHCO_3_, and 10 d-glucose and bubbled with the mixture of 95 % O_2_–5 % CO_2_. Coronal, frontal cortical slices (thickness 400 μm) containing a part of M1 were cut from one of the hemispheres between 3.5 and 1.7 mm rostral to bregma using a vibrating microtome (Leica VT1000). Slices were stored submerged in ACSF at 30 ± 0.5 °C. Primary motor cortex (3.5–1.7 mm rostral to bregma and 2.0–3.5 mm lateral to the midline) from the other hemisphere was dissected on ice and cryopreserved at −80 °C for later Western blotting analysis. In order to exclude the influence of lateralization, the brain hemispheres were used alternately for electrophysiology and Western blotting.

### Whole-cell recording

Individual slices were placed in the recording chamber mounted on the stage of the Zeiss Axio Examiner. D1 microscope and superfused at 3 ml/min with warm (32 ± 0.5 °C), modified ACSF of the following composition (in mM): 132 NaCl, 2 KCl, 1.25 KH_2_PO_4_, 26 NaHCO_3_, 1.3 MgSO_4_, 2.5 CaCl_2_, and 10 d-glucose 10, bubbled with 95 % O_2_–5 % CO_2_. Recording pipettes were pulled from borosilicate glass capillaries (Harvard Apparatus) using the Sutter Instrument P97 puller. The pipette solution contained (in mM): 130 K-gluconate, 5 NaCl, 0.3 CaCl_2_, 2 MgCl_2_, 10 HEPES, 5 Na_2_-ATP, 0.4 Na-GTP, 1 EGTA, and 0.1 % biocytin (osmolarity 290 mOsm, pH 7.2). Pipettes had open tip resistance of approx. 6 MΩ. Pyramidal cells were sampled from sites located at least 2 mm lateral to the midline and approx. 0.3 mm below the pial surface and were identified, as described previously [45]. Signals were recorded using the SEC 05LX amplifier (NPI, Germany), filtered at 2 kHz, and digitized at 20 kHz using Digidata 1440A interface and Clampex 10 software (Molecular Devices, USA).

### Electrophysiological characteristics of neurons

**Fig. 1:**
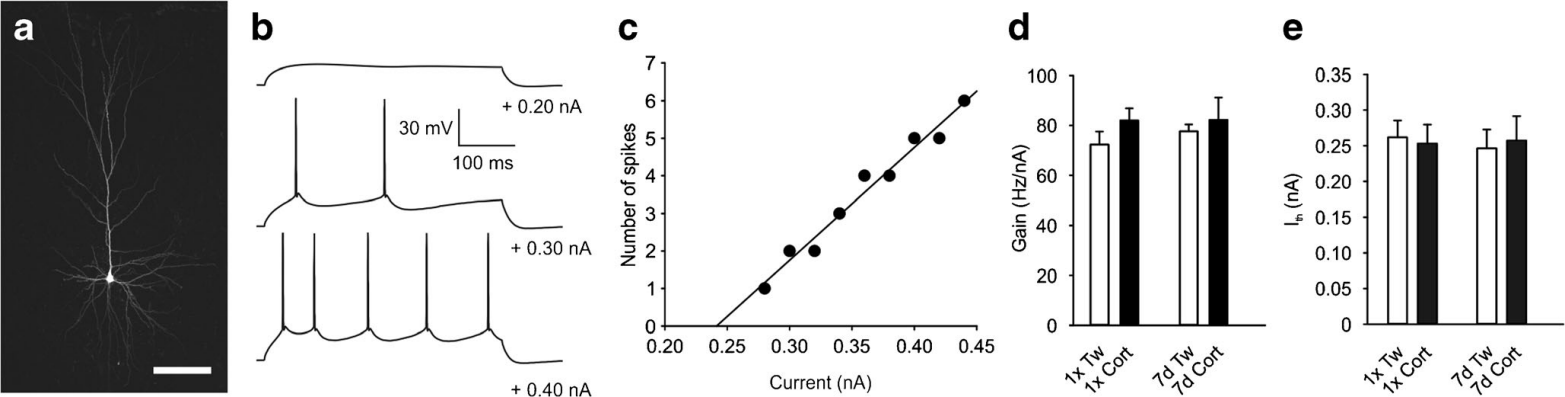
Corticosterone administration does not influence the intrinsic excitability of layer II/III pyramidal neurons. **a** Representative image showing biocytin-filled pyramidal cell after patch-clamp recording; *scale bar* = 100 μm. **b** Responses of a representative cell to sub- and suprathreshold depolarizing current pulses. **c** Graph of the number of action potentials vs. injected current for the cell shown in **b**. The threshold current (I_th_) corresponds to the extrapolated current at zero firing rate. The slope of the straight line fitted to experimental data represents gain. **d** Mean (±SEM) gain for control neurons (*Tw*) and neurons from corticosterone-treated animals (*Cort*), receiving injections once (*1×*) or repeatedly for 7 days (*7d*). **e** Mean (±SEM) threshold current. Labels as in **d**

After obtaining the whole-cell configuration and subsequent 10-min stabilization period, the firing characteristics of the recorded cells were assessed using intracellular injections of rectangular current pulses of increasing amplitude (duration 400 ms, Fig. 1b) in the current clamp mode. For each cell, the relationship between injected current intensity and the number of spikes was plotted. The gain was determined as a slope of the straight line fitted to experimental data (Fig. 1c; cf. [3]). The threshold current (I_th_) was determined as a current extrapolated at zero firing rate (Fig. 1c).

### Recording and analysis of sEPSCs and sIPSCs

**Fig. 2:**
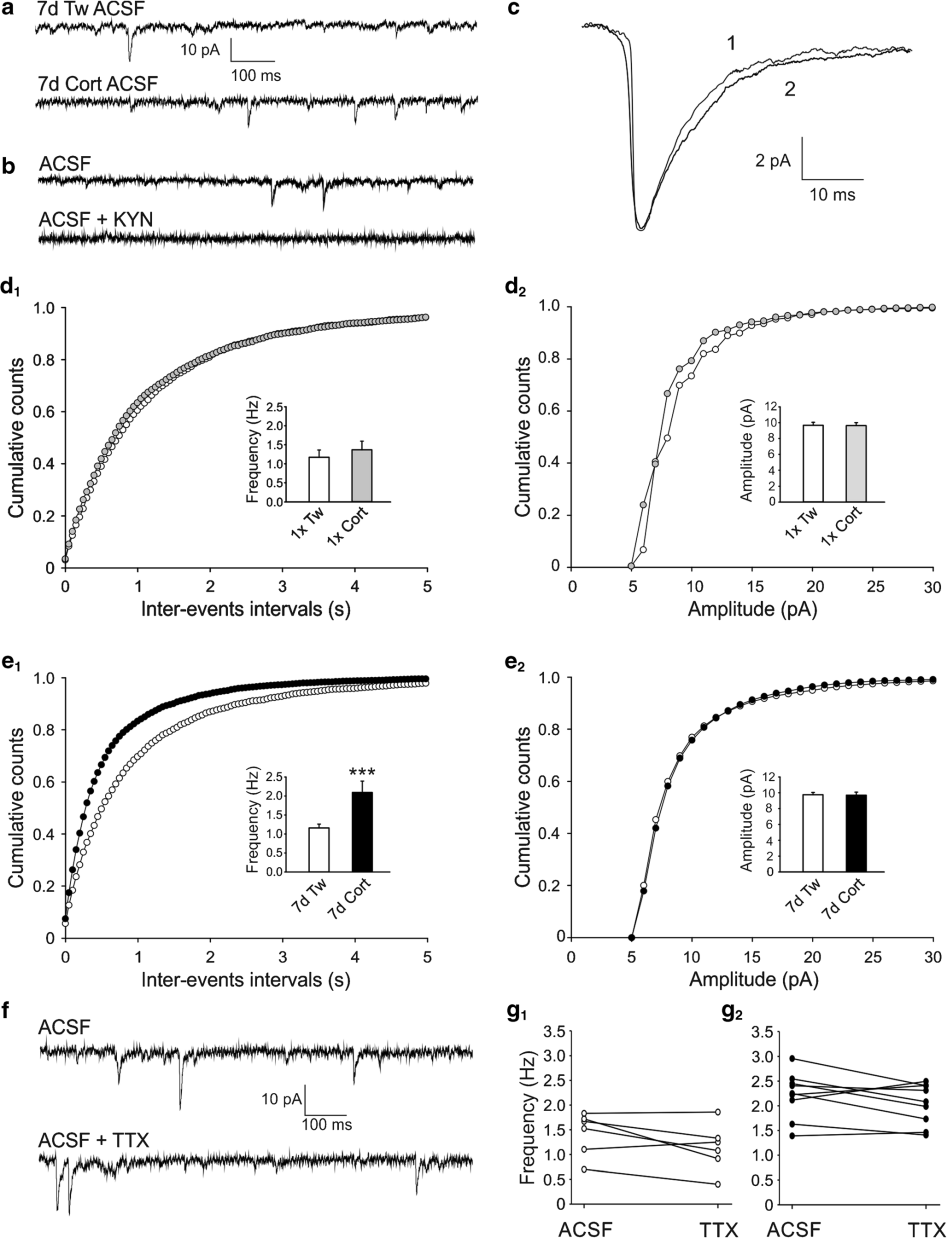
Repeated corticosterone-induced increase in the frequency of sEPSCs in layer II/III pyramidal cells. **a** Sample recordings in the ACSF of sEPSCs from representative neurons in slices prepared from the animal receiving vehicle (*Tw*, *upper trace*) and corticosterone for 7 days (*Cort*, *lower trace*). **b** Sample recordings from representative neurons in a slice prepared from control animal before (*upper trace*) and after (lower trace) addition of 2 mM kynurenic acid (*KYN*) to the ACSF. **c** Averaged sEPSCs, recorded over a period of 4 min from cells shown in a (*1*—Tween; *2*—corticosterone-treated). **d**_*1*_–**e**_*2*_ Averaged cumulative histograms of inter-event intervals (±SEM) and amplitudes of sEPSCs recorded from control cells (Tw, *open circles*) and from neurons from corticosterone-treated rats (Cort, *filled circles*) receiving injections once (*1×*, **d**_*1*_, **d**_*2*_) or repeatedly (*7d*, **e**_*1*_, **e**_*2*_). *Insets*—bar graphs illustrating a comparison of the mean frequency and the mean amplitude (± SEM) of sEPSCs recorded from neurons from control (*white bars*) and corticosterone-treated rats; once (*1×*—*gray bars*) or repeatedly (*7d*—*black bars*). ***p < 0.001. **f** Sample recordings of sEPSCs from a representative neuron in slice prepared from the animal receiving corticosterone for 7 days. *Upper trace*—recording in standard ACSF and in the presence of TTX (*lower trace*). **g**_*1*_, **g**_*2*_ Mean frequency of sEPSCs in individual neurons before and after addition of TTX to the ACSF in slices prepared from control (**g**_*1*_) and corticosterone-treated (**g**_*2*_) rats

**Fig. 3:**
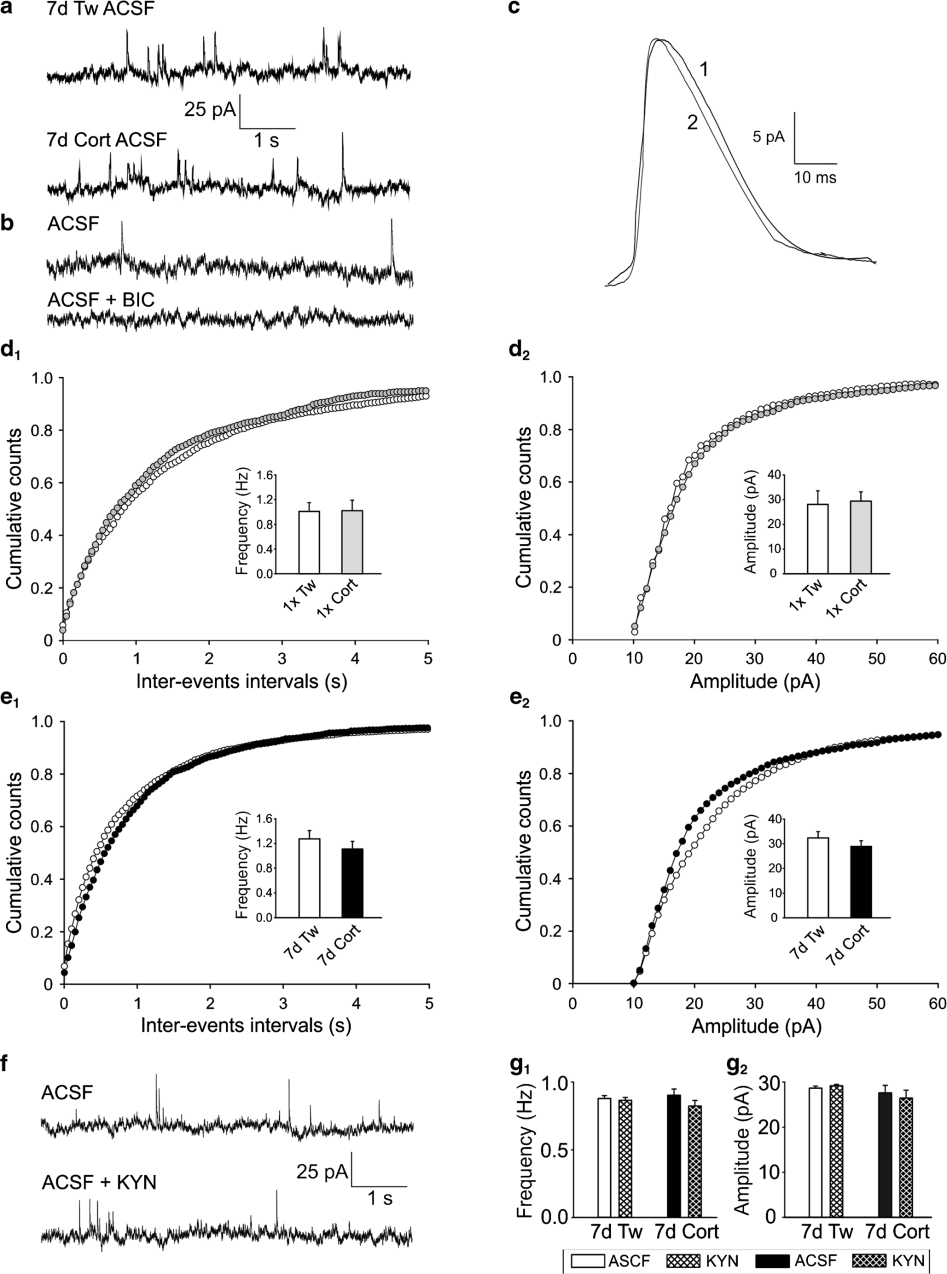
Lack of the effect of corticosterone on sIPSCs in layer II/III pyramidal cells. **a** Sample recordings of sIPSCs from representative neurons in slices prepared from the animal receiving vehicle (*upper trace*) or corticosterone for 7 days (*lower trace*). **b** Sample recordings from representative neurons in a slice prepared from control animal before (*upper trace*) and after addition of 20 μM bicuculline (BIC) to the ACSF (*lower trace*). **c** Averaged sIPSCs, recorded over a period of 4 min, from cells shown in a (*1*—Tween; *2*—corticosterone-treated). **d**_*1*_, **e**_*1*_ Averaged cumulative histograms of inter-event intervals (±SEM) of sIPSCs recorded in control cells (Tw, *open circles*) and in neurons from corticosterone-treated rats (Cort, *filled circles*) receiving injections once (*1×*) or repeatedly for 7 days (*7d*). **d**_*2*_–**e**_*2*_ Averaged cumulative histograms of amplitudes (±SEM) of sIPSCs. Labels as in **d**_*1*_, **e**_*1*_. *Insets* in **d**_*1*_–**e**_*2*_—bar graphs illustrating a comparison of the mean frequency and the mean amplitude (± SEM) of sIPSCs recorded from neurons from control (*white bars*) and corticosterone treated rats; once (*1×*—*gray bars*) or repeatedly (*7d*—*black bars*). **f** Sample recording of sIPSCs from a representative neuron in a slice prepared from control animal before (*upper trace*) and after (lower trace) addition of 2 mM kynurenic acid to the ACSF. **g**_*1*_ Mean frequency (± SEM) of sIPSCs recorded from individual neurons before and after addition of 2 mM kynurenic acid (*KYN*) to the ACSF in slices prepared from control (*7d Tw*) and corticosterone-treated (*7d Cort*) rats. The differences are not statistically significant. **g**_*2*_ Mean amplitude (± SEM) of sIPSCs recorded in individual neurons before and after addition of 2 mM kynurenic acid (*KYN*) to the ACSF. Labels as in **g**_*1*_

To record spontaneous EPSCs, neurons were voltage-clamped at −76 mV and synaptic events were recorded for 4 min as inward currents (Fig. 2a, b). Next, cells were voltage-clamped at 0 mV and after 15 min of stabilization period, sIPSCs were recorded for 4 min as outward currents (Fig 3a, b) [44]. This approach allowed us to record from the same neuron without a need to change the recording micropipette solution. Since this method requires high amplitude current injections, what results in a greater noise level, the smaller number of sIPSCs than sEPSCs recordings was suitable for further analysis. In order to block excitatory synaptic transmission, in a separate set of experiments, sIPSCs were recorded in the ACSF supplemented with 2 mM kynurenic acid.

Spontaneous EPSCs and IPSCs were detected offline using the automatic detection protocol (Mini Analysis software, Synaptosoft Inc.) and subsequently checked manually for accuracy. Data were accepted for the analysis when the access resistance ranged between 15 and 18 MΩ, and it was stable (<25 % change) during recording. The threshold amplitude for the detection of an EPSC was set at 5 pA, and for IPSCs, the detection threshold was set at 10 pA.

### Imaging and deconvolution

Following whole-cell patch clamp recordings with biocytin filling of recorded cells, slices were fixed for 24 h in 4 % formaldehyde in PBS. After several washings, tissue was incubated in phosphate-buffered saline containing 0.6 % Triton X-100 (Sigma-Aldrich) for 24 h and subsequently with 0.3 % Triton X-100 and Cy3-conjugated ExtrAvidin (1:200; Sigma-Aldrich) for 48 h. Slices were washed and mounted on glass slides, coverslipped with Vectashield containing DAPI (Vector Laboratories), and examined using the Zeiss LSM510 META confocal microscope (Microimaging GmbH, Jena, Germany). The selection scheme of dendritic segments for the spine counting was planned to minimize any possible bias. The imaging conditions were the following: 543-nm excitation wavelength generated by HeNe green laser attenuated to 60 % of its full power with Acusto-Optical Filter (AOF), 488/543 dichroic mirror to reflect excitation light to the oil immersion 63×/1.4 Plan Apochromat objective (Zeiss, Germany), 560 LP emission filter, and PMT with gain and offset adjusted to image the specimens without the clipping effect. The set of optical slices was taken for each specimen at pinhole aperture open to 1 Airy unit (106 μm pinhole diameter), 35 μs pixel time, and 604 × 604 pixels of image resolution under the 5× digital zoom to obtain Nyquist sampling criterion—*X* = 47 nm, *Y* = 47 nm, and *Z* = 160 nm. The experimental point spread function (PSF) for the microscope optical arrangement was calculated using images of 0.175-μm fluorescent beads (PS-Speck Microscope Point Source Kit (P7220), Life Technologies, USA). Huygens Professional software (version 4.2, SVI, Netherlands) was used for image deconvolution. PSF was calculated on the basis of at least 25 distilled and accumulated images of the beads followed by object stabilizer approach. Then, all optical sections of fluorescently tagged cells imaged in the confocal microscope (see above) were deconvolved at signal-to-noise ratio (SNR) value set to 20. Finally, the images were saved for manual spine counting from the optical stacks. Every protrusion considered to be a spine was verified on the basis of a 3D projection constructed from the stack in ImageJ (NIH). Data analyses were performed by an experimenter blinded to the treatment condition.

### Western blotting

The GluA1 and GluA2 subunits were chosen for the analysis since it has been estimated that more than 80 % of native AMPA receptors are GluA1A2 heteromers [26, 35]. For NMDA and GABA_A_ receptors, we chose GluN1; GluN2A; GluN2B; and α_1_, β_2_, γ_2_ subunits, respectively, since synaptic receptors containing these subunits are most abundant in the cortex [6, 17, 27].

Cortical tissue samples were thawed and homogenized with Ultra Turrax T25 (Janke & Kunkel IKA-47 Labortechnik) in 300 μl of ice-cold solution of RIPA buffer (Sigma-Aldrich) containing phenylmethane–sulfonyl fluoride, sodium orthovanadate, Protease Inhibitor Cocktail, Phosphatase Inhibitor Cocktails 1 and 2 (Sigma-Aldrich), and 5 % glycerol. The homogenate was centrifuged (14,000×*g*, 4 °C) for 20 min to obtain the crude membrane fraction. Protein concentration was determined using the colorimetric technique and BCA protein assay kit (Pierce, Rockford, IL). A standard curve was generated and then absorbance of samples was measured with the Multiskan Spectrum microplate spectrophotometer (Thermo Labsystems). Supernatants from all the samples were diluted with RIPA buffer to obtain the same protein concentration in 150 μl of the solution. Samples were boiled for 7 min at 99 °C in Laemmli (Bio-Rad) sample buffer with 2-mercaptoethanol (Sigma-Aldrich). Protein samples were analyzed by Western blotting, as described previously [48]. Afterwards, the membranes were incubated with affinity-purified polyclonal antibodies against AMPA, NMDA, and GABA_A_ receptor subunits: GluA1 and GluA2 (1:1000, rabbit; Millipore), GluN1, GluN2A, GluN2B (1:200; goat; Santa Cruz Biotechnology Inc.) in 3 % non-fat dry milk–TBST as well as GABA_A_ receptor subunits: α_1_ (1:200, goat; Santa Cruz Biotechnology Inc.) and β_2_, γ_2_ (1:1000, rabbit; Millipore) in 1 % bovine serum albumin (BSA)–TBST at 4 °C overnight. On the next day, membranes were incubated with horseradish peroxidase-conjugated secondary antibody (1:2000, Vector Laboratories) in 3 % non-fat dry milk or 1 % BSA–TBST, for 60 min at room temperature. The secondary antibody used for the GluN1, GluN2A, GluN2B, and α_1_ subunit was an anti-goat antibody, whereas the secondary antibody used for the GluA1, GluA2, β_2_, and γ_2_ subunits was an anti-rabbit antibody. Proteins were detected using a chemiluminescent method (Immun-Star HRP). Results were normalized to GAPDH (36 kDa), using a GAPDH loading control mouse antibody (1:1000; Thermo Fisher Scientific Inc.).

### Statistics

Statistical analysis of electrophysiological data was carried out using two-way ANOVA with post hoc Holm–Sidak method and post hoc Tukey test for the assessment of the longevity of corticosterone effects. Cumulative inter-event interval and amplitude distributions of postsynaptic events were compared using the Kolmogorov–Smirnov (KS) test. Western blotting and spine density data were evaluated using Student’s *t* test. Rats were weighted on a daily basis, and body mass was analyzed using repeated measures two-way ANOVA. *p* < 0.05 was considered as statistically significant.

## Results

### Corticosterone and neuronal excitability

Control and corticosterone-treated rats had similar body mass after single and after repeated administration (1× Tween, 142.5 ± 9.7 g; 1× Cort, 131.0 ± 9.3 g; *t* (17) = 0.754, *p* = 0.462; and 7d Tween, 190.9 ± 9.2 g; 7d Cort, 171.0 ± 9.3 g; *t* (17) = 1.532, *p* = 0.144, respectively).

All recorded cells exhibited a regular spiking firing pattern in response to a depolarizing current pulse, typical of pyramidal neurons (Fig. 1b). There were no statistically significant differences between neurons originating from corticosterone-treated groups of animals and respective control cells either in the resting membrane potential or in the input resistance (Table 1). To assess the intrinsic excitability, the relationship between the firing rate and the intensity of the injected current was evaluated for each neuron (Fig. 1c). As illustrated in Fig. 1d, e, neither the mean gain nor the mean threshold value of action potential generation differed between experimental and control groups, indicating that corticosterone treatment did not influence the excitability of layer II/III pyramidal neurons.

**Table 1:** Basic parameters of recorded neurons (mean ± SEM)

Group	V_m_ (mV)	R_m_ (MΩ)	*n*
1× Tween	−70.85 ± 1.27	47.46 ± 4.29	13
1× Corticosterone	−71.27 ± 0.78	58.58 ± 6.29	15
7d Tween	−69.39 ± 1.32	50.59 ± 6.81	19
7d Corticosterone	−69.00 ± 1.04	56.85 ± 6.18	19

Differences between values for neurons in experimental (corticosterone) and control (Tween) groups, receiving injections once (1×) or repeatedly for 7 days (7d) are not significant (*p* > 0.05).

*V*_*m*_ resting membrane potential, *R*_*m*_ input resistance, *n* number of cells

### Effects of corticosterone on sEPSCs and sIPSCs

A single injection of corticosterone affected neither the mean frequency nor the mean amplitude of sEPSCs (Fig. 2d_*1*_, d_*2*_; Table 2). The occurrence of spontaneous EPSCs could be blocked by 2 mM kynurenic acid (Fig. 2b). Amplitudes and inter-event interval times of sEPSCs were plotted as cumulative distribution histograms (Fig. 2d–e). Cumulative distribution of both amplitudes and inter-event interval intervals of sEPSCs did not differ between control and corticosterone-treated groups (KS test, *p* > 0.05). However, in slices originating from rats treated with corticosterone for 7 days and prepared 2 days after the last hormone administration, the mean frequency, but not the mean amplitude of sEPSCs, was markedly higher than that in the cells originating from control animals receiving vehicle (Fig. 2e_*1*_, e_*2*_; Table 2). These findings were validated by comparing data from the respective cumulative distribution histograms of sEPSCs frequencies (KS test, *p* < 0.001, Fig. 2e_*1*_) and amplitudes (KS test, *p* = 0.8, Fig. 2e_*2*_). The rise time and the decay time constant of the averaged sEPSCs were similar in both groups (Fig. 2c, Table 2).

**Table 2:** Effects of corticosterone treatment on parameters characterizing sEPSCs (mean ± SEM)

Group	Mean frequency (Hz)	Mean amplitude (pA)	Rise time (ms)	Decay time constant (*τ*, ms)	*n*
1× Tween	1.17 ± 0.19	9.66 ± 0.38	2.75 ± 0.14	10.71 ± 0.76	14
1× Corticosterone	1.36 ± 0.23	9.63 ± 0.38	2.68 ± 0.12	10.36 ± 0.78	15
7d Tween	1.16 ± 0.10	9.76 ± 0.28	2.80 ± 0.08	10.13 ± 0.48	19
7d Corticosterone	2.09 ± 0.30 ***	9.68 ± 0.39	2.64 ± 0.13	9.68 ± 0.44	19

***p < 0.001 indicates a significant difference between experimental (corticosterone) and control (Tween) groups, receiving injections once (1×) or repeatedly for 7 days (7d)

*n* number of cells

To test the contribution of action potential-related glutamate release to recorded sEPSCs, the effect of Na^+^ channel blocker tetrodotoxin (TTX) was investigated in a separate sample of 6 control and 9 neurons obtained from corticosterone-treated (7d) animals (Fig. 2f). The addition of 0.5 μM TTX to the ACSF did not change either the mean frequency (Tween, 1.43 ± 0.18 Hz; in TTX, 1.14 ± 0.20 Hz; *t* (5) = 2.061, *p* = 0.094; see Fig. g_*1*_, and Cort 2.22 ± 0.16 Hz; in TTX, 2.03 ± 0.14 Hz; *t* (8) = 1.634, *p* = 0.141; see Fig. 2g_*2*_) or the mean amplitude (Tween, 14.26 ± 0.89 pA; in TTX, 12.27 ± 0.59 pA; *t* (5) = 1.570, *p* = 0.096, and Cort 13.82 ± 0.56 pA; in TTX, 12.91 ± 0.48 pA; *t* (8) = 1.362, *p* = 0.21) of sEPSCs. Thus, it is likely that most of sEPSCs recorded in the present study correspond to miniature EPSCs (mEPSCs).

Corticosterone administration had no effect on parameters characterizing spontaneous IPSCs, which could be blocked by 20 μM bicuculline (Fig. 3b). Both the frequency and amplitude (Fig. 3d_*1*_–e_*2*_) as well as the rise time and the decay time constant of sIPSCs (Fig. 3c, Table 3) were not significantly different from control values in any of the experimental groups. Concordantly, corticosterone treatment altered neither the inter-event interval (Fig. 3e_*1*_, e_*2*_, KS test, *p* = 0.5) nor the amplitude (Fig. 3e_*1*_, e_*2*_, KS test, *p* = 0.9) distribution of sIPSCs.

**Table 3:** Effects of corticosterone treatment on parameters characterizing sIPSCs (mean ± SEM)

Group	Mean frequency (Hz)	Mean amplitude (pA)	Rise time (ms)	Decay time constant (*τ*, ms)	*n*
1× Tween	1.01 ± 0.14	28.11 ± 5.50	3.22 ± 0.17	12.48 ± 2.10	10
1× Corticosterone	1.02 ± 0.17	29.45 ± 3.78	2.69 ± 0.37	14.53 ± 1.87	6
7d Tween	1.28 ± 0.13	32.41 ± 2.52	2.54 ± 0.16	17.76 ± 1.25	13
7d Corticosterone	1.11 ± 0.12	28.85 ± 2.32	2.69 ± 0.16	15.50 ± 1.26	8

Differences between values for experimental (corticosterone) and control (Tween) groups, receiving injections once (1×) or repeatedly for 7 days (7d) are not significant (*p* > 0.05)

*n* number of cells

To exclude the possibility that sEPSCs contributed to spontaneous synaptic activity recorded at 0 mV, in a separate set of recordings (9 cells originating from 4 rats receiving Tween for 7 days, 10 cells from 5 rats receiving corticosterone for 7 days), 2 mM kynurenic acid was added to the ACSF (Fig. 3f). As illustrated in Fig. 3g_*1*_, g_*2*_, neither the mean frequency nor the mean amplitude of postsynaptic events recorded from neurons originating both from corticosterone-treated and control animals was affected by the blockade of glutamatergic transmission.

### Corticosterone and the density of dendritic spines

**Fig. 4:**
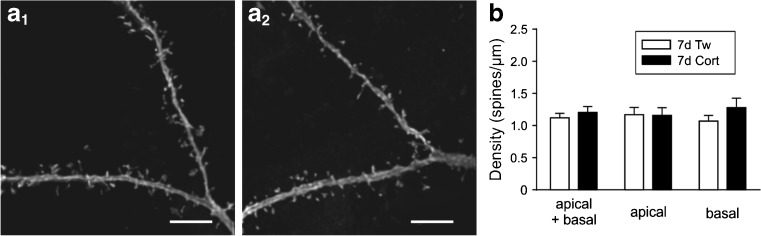
Repeated administration of corticosterone does not change the density of dendritic spines in layer II/III pyramidal cells. **a**_*1*_, **a**_*2*_ Representative images of dendritic segments of neurons originating from a control rat (**a**_*1*_) and an animal receiving corticosterone for 7 days (**a**_*2*_); *scale bars* = 10 μm. **b** Corticosterone (*Cort*) treatment lasting 7 days (*7d*) did not affect the density of spines on total (apical + basal), apical nor basal dendrites compared to neurons originating from rats receiving vehicle (*7d Tw*). Spine density is defined as the mean number of spines per micrometer of dendrite length. The differences are not statistically significant

For the dendritic spine counting, 11 neurons originating from rats treated with corticosterone for 7 days and 9 cells obtained from control animals were analyzed. The dendritic spines were counted on the total length of 1961.5 μm on 43 independent secondary, tertiary, and quaternary dendritic branch segments from the corticosterone group and on 1202.5 μm on 32 segments from the control group, within 10–90 μm from the soma. In total, 2496 spines on cortical neurons from corticosterone-treated animals and 1379 spines on cortical neurons from control animals were analyzed. The overall spine density did not differ significantly between the groups (1.2 ± 0.09 vs. 1.12 ± 0.07 spines/μm, respectively; *p* = 0.5, Fig. 4). No significant differences in spine density of basal dendrites were found between the groups (1.3 ± 0.15 vs. 1.07 ± 0.09 spines/μm, respectively; *p* = 0.2). The density of spines was also similar in the apical dendritic tree (1.6 ± 0.12 vs. 1.17 ± 0.11 spines/μm, respectively; *p* = 0.9).

### Corticosterone and AMPA, NMDA, and GABA_A_ receptor subunits

**Fig. 5:**
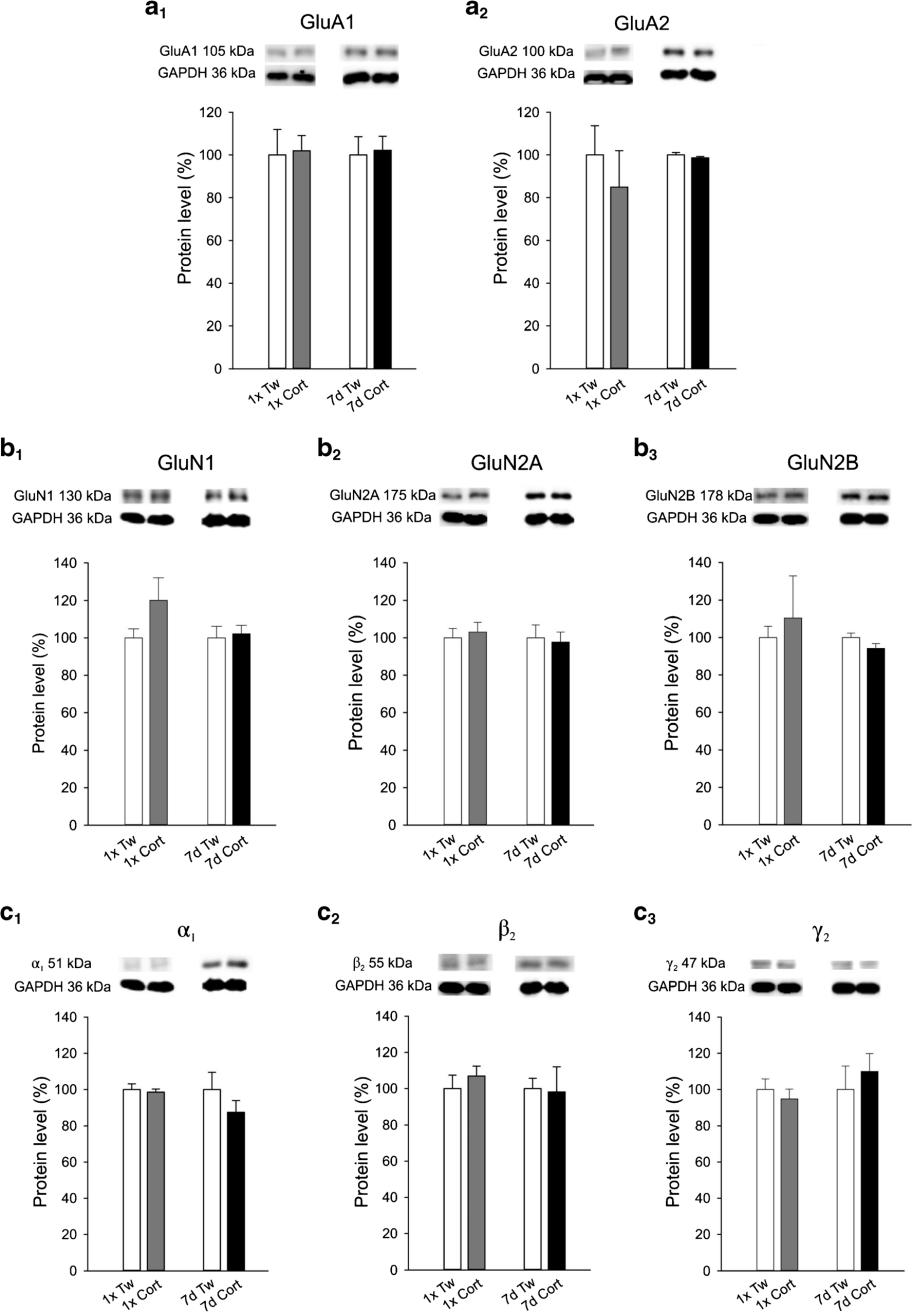
Western blot analysis of AMPA, NMDA, and GABA_A_ receptor subunit proteins in the M1. *Bar graphs* represent summary results of the densitometric analysis of subunit proteins from the motor cortex of rats treated with corticosterone for 7 days (mean ± SEM; *black bars*) with respective controls, receiving vehicle (*white bars*). The differences are not statistically significant. In each bar, *n* = 6–8. The *above bar graphs* show computerized scans of representative Western immunoblots illustrating subunit protein bands in corticosterone-treated and control groups as well as GAPDH bands

Repeated treatment with corticosterone did not alter protein levels of GluA1 and GluA2 subunits of the AMPA receptor (Fig. 5a_*1*_–a_*2*_). Also protein levels of GluN1, GluN2A, and GluN2B subunits of the NMDA receptor (Fig. 5b_*1*_–b_*3*_) as well as that of α_1_, β_2_, and γ_2_ subunits of the GABA_A_ receptor (Fig. 5c_*1*_–c_*3*_) were not affected.

### Longevity of the corticosterone-induced effect

**Fig. 6:**
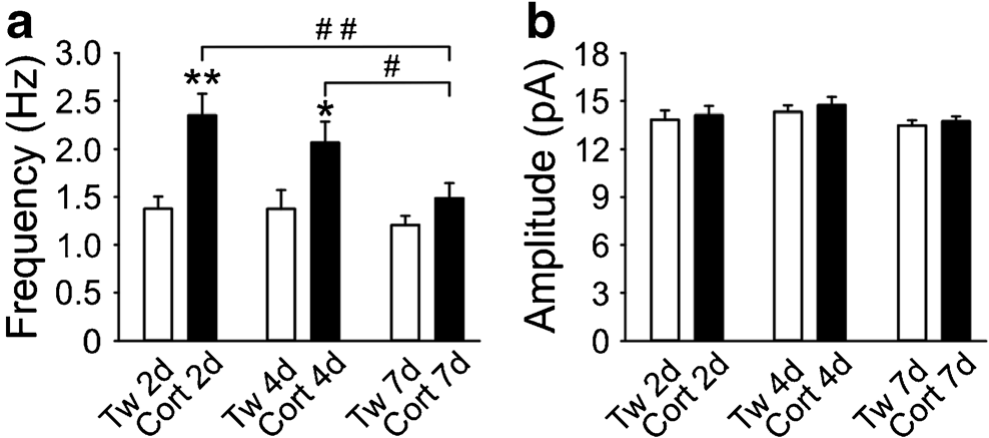
Longevity of corticosterone-induced effect on the frequency of sEPSCs in layer II/III M1 pyramidal neurons. **a** The mean frequency of the sEPSCs recorded 2, 4, or 7 days after last drug administration from neurons obtained from animals treated with Tween or corticosterone for 7 days. *, #*p* < 0.05; **, ##*p* < 0.01. **b** The mean amplitude of the sEPSCs recorded 2, 4, or 7 days after last drug administration from neurons from animals treated with Tween or corticosterone for 7 days

In a separate group of animals, the mean frequency and amplitude of sEPSCs were compared between slices prepared 2, 4, or 7 days after the end of the corticosterone treatment lasting for 7 days. As illustrated in Fig. 6a, the mean frequency of sEPSCs measured in slices prepared 4 days after the end of the treatment was not significantly different from that measured 2 days after the treatment ended. In contrast, in slices prepared 7 days after the last corticosterone administration, the mean frequency of the sEPSCs was not different from the control, receiving the vehicle (*p* = 0.236). In all cases, the mean amplitude of sEPSCs did not deviate from control (Fig. 6b). The analysis did not reveal a significant effect of treatment [*F* = 0.753; *p* = 0.388], time [*F* = 2.239; *p* = 0.112], nor significant interaction between time and treatment [*F* = 0.0201; *p* = 0.98].

## Discussion

We have previously reported that glutamatergic field potentials evoked in layer II/III of the frontal cortical area M1 by stimulation of underlying sites were increased after repeated corticosterone administration lasting 7 and 21 days. Corticosterone treatment has been found to increase the mean frequency, but not the mean amplitude, of sEPSCs in a sample of layer II/III pyramidal neurons [4]. In the present study, we have extended those findings by showing that corticosterone treatment-induced increase in the frequency of sEPSCs is absent after a single injection of the hormone when tested 2 days later and, moreover, in the same population of neurons, neither single nor repeated corticosterone injections affected sIPSCs. We also show that the sodium channel blocker tetrodotoxin does not significantly change the mean frequency of sEPSCs in layer II/III pyramidal cells of the M1 area, indicating that most of the recorded spontaneous currents correspond to action potential-independent miniature EPSCs (mEPSCs; this study and [45; 48]). The observed increase in the mean frequency of sEPSCs is consistent with a presynaptic mechanism of the effect, related to an enhanced spontaneous release of glutamate quanta from presynaptic terminals. This conclusion is supported by a lack of changes in the protein level of the tested subunits of postsynaptic ionotropic receptors as well as with a lack of changes in the density of dendritic spines. Present data also demonstrate that membrane excitability of the recorded cells remains unchanged when tested 2 days after the end of corticosterone treatment and are consistent with the observation that direct effects of corticosterone on neuronal excitability decline to baseline values within hours [16].

The influence of repeated stress or exogenous corticosterone treatment on synaptic transmission in the rat M1 has not been investigated previously. Earlier studies aimed at finding cellular and molecular mechanisms underlying the influence of stress on cortical glutamatergic transmission have mainly been focused on the medial prefrontal cortex. It was demonstrated that restraint stress (2 h/day) and unpredictable stress chosen from six stressors, when repeated for 5–7 days, induce a reduction in the amplitude of stimulation-evoked and miniature EPSCs [51]. Since these effects were accompanied by reduced protein levels of AMPA and NMDA receptor subunits but unchanged paired-pulse ratio of the evoked EPSCs, their mechanisms are mainly postsynaptic. These authors have also found that the frequency of mEPSCs was decreased, implying a contribution of a presynaptic mechanism. Another study has shown that 21 days of chronic unpredictable stress resulted in a reduction in both the frequency and the amplitude of sEPSCs, accompanied by a decrease in the expression level of several pre- and postsynaptic proteins [24]. On the other hand, Ito et al. [2010] have found no change in field potentials and mEPSCs in the anterior cingulate cortex of mice subjected to restraint lasting 2 h/day for 7 days. However, these authors observed a reduction in the frequency of IPSCs. The diverse outcomes of cited and our studies may result from the use of different experimental models but it is also likely that the responses of different cortical areas to stress are diverse. For example, while in the rat mPFC, the dendritic length and spine density decrease after stress, in the orbitofrontal cortex, they increase [22].

It has been reported that repeated corticosterone administration (40 mg/kg) for 21 days increased the immobility in the forced swim test, while repeated restraint stress had no effect on this depression-like behavior [11]. A lower dose of corticosterone (20 mg/kg per day), similar to that used in the present study, has also been found to increase immobility in the forced swim test [13]. The effects of repeated stress on the rat M1 have not yet been investigated; however, it is likely that they would be weaker than the effects of repeated corticosterone administration. The responses of the organism to repeated homotypic stress show the adaptive reduction, a phenomenon regarded as a form of habituation (reviewed in [12]). A contribution of the adaptation appears to be smaller in the unpredictable stress paradigms, however, it has been demonstrated by several authors that behavioral stress-induced corticosterone level increases are transient while repeated corticosterone administration-induced elevation of plasma corticosterone level lasts for about 24 h after the last injection [7, 11, 46]. Thus, repeated corticosterone injections are likely to exert a stronger influence on the plasma corticosterone level than repeated stress sessions. As a consequence, for example, repeated corticosterone-treated rats weigh less than the animals subjected to repeated restraint [28]. In our study after 7 days of treatment, the difference was not significant, which may be attributed to the selected dose of corticosterone (cf. [11]). Interestingly, our data show that despite a prolonged exposure to elevated levels of corticosterone, no changes were evident in the GABAergic input to layer II/III pyramidal neurons, in contrast to the glutamatergic one. On the other hand, decreased levels of GABA and GABAergic transmission have been found in the prefrontal cortex of animals subjected to chronic mild stress and chronic social defeat stress [14, 47]. Again, the use of not only a different experimental model but also different responses to corticosterone of different cortical areas are likely reasons of the apparent discrepancy.

Our data demonstrate that corticosterone treatment did not influence the protein levels of GluA1, GluA2, GluN1, GluN2A, and GluN2B subunits of ionotropic glutamate receptors or that of α_1_, β_2_, and γ_2_ subunits of the GABA_A_ receptor in the rat motor cortex. Other studies showed changes in the expression levels of some receptor subunits in other cortical areas. It has been reported that repeated corticosterone administration for 14 days resulted in a decrease in the expression level of GluN2B and GluA2/3 subunit proteins in the rat medial prefrontal cortex, but no changes were evident in either adjacent lateral orbitofrontal cortex or the hippocampus [10]. Regarding the GABAergic system, corticosterone-treated rats expressed altered protein level of the α_2_, but not other subunits of the GABA_A_ receptor in the hippocampus [28]. In contrast to the cortex, no effects of repeated stress on basal glutamatergic transmission were detected in the striatum, hippocampal CA1 area [51], and the dentate gyrus [18].

Several studies have shown that chronic treatment of rats with antidepressant drugs reduces excitatory synaptic transmission (reviewed in [31]). We have previously demonstrated that repeated administration of a tricyclic antidepressant imipramine, lasting for 2 weeks, attenuates glutamatergic transmission in the rat frontal cortex by decreasing the mean frequency and, to a lesser degree, the mean amplitude of sEPSCs [45]. Imipramine administration normalized repeated corticosterone-induced increase in the field potential amplitude and sEPSC frequency and, moreover, restored corticosterone-impaired potential for the induction of long-term potentiation in the frontal cortex [4]. These data suggest that the influence of imipramine on glutamatergic transmission is opposite to that of repeated corticosterone administration. Our earlier work also showed that imipramine administration counteracted repeated corticosterone-induced functional modifications in the reactivity of 5-HT_1A_ and 5-HT_2_ receptors in the rat frontal cortex [52].

Acute stress and single episodes of exposure to corticosterone transiently increase the release of glutamate from presynaptic terminals in the prefrontal and frontal cortex (reviewed in [34]). It is conceivable that repeated episodes of transient enhancement of excitatory transmission via pre- and/or postsynaptic mechanisms [50] may eventually induce a sustained modification of glutamate release via activity-dependent mechanisms.

Spontaneous excitatory postsynaptic events, previously regarded as mere synaptic noise, have been proposed to play a key role in a homeostatic regulation of synaptic efficacy by controlling dendritic protein synthesis [43]. It has been demonstrated that the level of spontaneous glutamate release adjusts the plasticity threshold at single synapses, thus representing a form of metaplasticity [23]. In line with these results, our previous study demonstrated that repeated corticosterone administration-induced enhancement of spontaneous excitatory transmission coincides with a reduced possibility for LTP induction in the rat motor cortex [4]. Of note, blockade of the NMDA component of spontaneous EPSCs has been implicated in a rapid antidepressant-like effect of ketamine and other NMDA receptor antagonists in animal models [2].

In summary, we demonstrate that repeated exogenous corticosterone administration to the adolescent rats results, by a currently unknown mechanism, in an increase in the frequency of sEPSCs, but not other parameters characterizing the glutamatergic input to layer II/III pyramidal neurons of the M1. Furthermore, we show that the GABAergic transmission remains unchanged after corticosterone treatment. These findings add to a growing body of evidence that chronically elevated corticosterone level results in a distortion of the balance between glutamate and GABA system in the brain.

In conclusion, we propose that corticosterone treatment evoked potentiation of glutamatergic transmission in the motor cortex and constitutes the potential mechanisms underlying detrimental influence of stress on skilled movement patterns and skilled movement accuracy in M1-dependent reaching and walking tasks [29].

## References

[1] Antonow-Schlorke I, Ehrhardt J, Knieling M (2013). Modification of the ladder walking task—new options for analysis of skilled movements. Stroke Res Treat.

[2] Autry AE, Adachi M, Nosyreva E, Na ES, Los MF, Cheng PF, Kavalali ET, Monteggia LM (2011). NMDA receptor blockade at rest triggers rapid behavioural antidepressant responses. Nature.

[3] Bekisz M, Garkun Y, Wabno J, Hess G, Wrobel A, Kossut M (2010). Increased excitability of cortical neurons induced by associative learning: an ex vivo study. Eur J Neurosci.

[4] Bobula B, Wabno J, Hess G (2011). Imipramine counteracts corticosterone-induced enhancement of glutamatergic transmission and impairment of long-term potentiation in the rat frontal cortex. Pharmacol Rep.

[5] Checkley S (1996). The neuroendocrinology of depression and chronic stress. Br Med Bull.

[6] Cull-Candy S, Brickley S, Farrant M (2001). NMDA receptor subunits: diversity, development and disease. Curr Opin Neurobiol.

[7] Czyrak A, Maćkowiak M, Chocyk A, Fijał K, Tokarski K, Bijak M, Wedzony K (2002). Prolonged corticosterone treatment alters the responsiveness of 5-HT_1A_ receptors to 8-OH-DPAT in rat CA1 hippocampal neurons. Naunyn Schmiedeberg’s Arch Pharmacol.

[8] Donoghue JP, Wise SP (1982). The motor cortex of the rat: cytoarchitecture and microstimulation mapping. J Comp Neurol.

[9] Gilabert-Juan J, Castillo-Gomez E, Guirado R, Moltó MD, Nacher J (2012). Chronic stress alters inhibitory networks in the medial prefrontal cortex of adult mice. Brain Struct Funct.

[10] Gourley SL, Kedves AT, Olausson P, Taylor JR (2009). A history of corticosterone exposure regulates fear extinction and cortical NR2B, GluR2/3, and BDNF. Neuropsychopharmacology.

[11] Gregus A, Wintink AJ, Davis AC, Kalynchuk LE (2005). Effect of repeated corticosterone injections and restraint stress on anxiety and depression-like behavior in male rats. Behav Brain Res.

[12] Grissom N, Bhatnagar S (2009). Habituation to repeated stress: get used to it. Neurobiol Learn Mem.

[13] Hill MN, Brotto LA, Lee TT, Gorzalka BB (2003). Corticosterone attenuates the antidepressant-like effects elicited by melatonin in the forced swim test in both male and female rats. Prog Neuropsychopharmacol Biol Psychiatry.

[14] Ito H, Nagano M, Suzuki H, Murakoshi T (2010). Chronic stress enhances synaptic plasticity due to disinhibition in the anterior cingulate cortex and induces hyper-locomotion in mice. Neuropharmacology.

[15] Jadavji NM, Supina RD, Metz GA (2011). Blockade of mineralocorticoid and glucocorticoid receptors reverses stress-induced motor impairments. Neuroendocrinology.

[16] Joëls M (2011). Impact of glucocorticoids on brain function: relevance for mood disorders. Psychoneuroendocrinology.

[17] Johnstone GA (2005). GABA_A_ receptor channel pharmacology. Curr Pharm Des.

[18] Karst H, Joëls M (2003). Effect of chronic stress on synaptic currents in rat hippocampal dentate gyrus neurons. J Neurophysiol.

[19] Kleim JA, Barbay S, Nudo RJ (1998). Functional reorganization of the rat motor cortex following motor skill learning. J Neurophysiol.

[20] Kleim JA, Lussnig E, Schwarz ER, Comery TA, Greenough WT (1996). Synaptogenesis and Fos expression in the motor cortex of the adult rat after motor skill learning. J Neurosci.

[21] Kirkland SW, Smith L, Metz GA (2012). Task-specific compensation and recovery following focal motor cortex lesion in stressed rats. J Integr Neurosci.

[22] Kolb B, Gibb R (2015). Plasticity in the prefrontal cortex of adult rats. Front Cell Neurosci.

[23] Lee MC, Yasuda R, Ehlers MD (2010). Metaplasticity at single glutamatergic synapses. Neuron.

[24] Li N, Liu RJ, Dwyer JM, Banasr M, Lee B, Son H, Li XY, Aghajanian G, Duman RS (2011). Glutamate N-methyl-D-aspartate receptor antagonists rapidly reverse behavioral and synaptic deficits caused by chronic stress exposure. Biol Psychiatry.

[25] Lopez JF, Chalmers DT, Little KY, Watson SJ (1998). Regulation of serotonin1A, glucocorticoid, and mineralocorticoid receptor in rat and human hippocampus: implications for the neurobiology of depression. Biol Psychiatry.

[26] Lu W, Shi Y, Jackson AC, Bjorgan K, During MJ, Sprengel R, Seeburg PH, Nicoll RA (2009). Subunit composition of synaptic AMPA receptors revealed by a single-cell genetic approach. Neuron.

[27] Luscher B, Keller CA (2004). Regulation of GABA_A_ receptor trafficking, channel activity, and functional plasticity of inhibitory synapses. Pharmacol Ther.

[28] Lussier AL, Romay-Tallón R, Caruncho HJ, Kalynchuk LE (2013). Altered GABAergic and glutamatergic activity within the rat hippocampus and amygdala in rats subjected to repeated corticosterone administration but not restraint stress. Neuroscience.

[29] Metz GA, Jadavji NM, Smith LK (2005). Modulation of motor function by stress: a novel concept of the effects of stress and corticosterone on behavior. Eur J Neurosci.

[30] Morimoto M, Morita N, Ozawa H, Yokoyama K, Kawata M (1996). Distribution of glucocorticoid receptor immunoreactivity and mRNA in the rat brain: an immunohistochemical and in situ hybridization study. Neurosci Res.

[31] Musazzi L, Treccani G, Mallei A, Popoli M (2013). The action of antidepressants on the glutamate system: regulation of glutamate release and glutamate receptors. Biol Psychiatry.

[32] Nudo RJ, Jenkins WM, Merzenich MM (1990). Repetitive microstimulation alters the cortical representation of movements in adult rats. Somatosens Mot Res.

[33] Parker KJ, Schatzberg AF, Lyons DM (2003). Neuroendocrine aspects of hypercortisolism in major depression. Horm Behav.

[34] Popoli M, Yan Z, McEwen BS, Sanacora G (2011). The stressed synapse: the impact of stress and glucocorticoids on glutamate transmission. Nat Rev Neurosci.

[35] Reimers JM, Milovanovic M, Wolf ME (2011). Quantitative analysis of AMPA receptor subunit composition in addiction-related brain regions. Brain Res.

[36] Remple MS, Bruneau RM, VandenBerg PM, Goertzen C, Kleim JA (2001). Sensitivity of cortical movement representations to motor experience: evidence that skill learning but not strength training induces cortical reorganization. Behav Brain Res.

[37] Rioult-Pedotti MS, Donoghue JP, Dunaevsky A (2007). Plasticity of the synaptic modification range. J Neurophysiol.

[38] Rioult-Pedotti MS, Friedman D, Donoghue JP (2000). Learning-induced LTP in neocortex. Sci (Wash).

[39] Rioult-Pedotti MS, Friedman D, Hess G, Donoghue JP (1998). Strengthening of horizontal cortical connections following skill learning. Nat Neurosci.

[40] Roland BL, Krozowski ZS, Funder JW (1995). Glucocorticoid receptor, mineralocorticoid receptors, 11 beta-hydroxysteroid dehydrogenase-1 and -2 expression in rat brain and kidney: in situ studies. Mol Cell Endocrinol.

[41] Shansky RM, Morrison JH (2009). Stress-induced dendritic remodeling in the medial prefrontal cortex: effects of circuit, hormones and rest. Brain Res.

[42] Sterner EY, Kalynchuk LE (2010). Behavioral and neurobiological consequences of prolonged glucocorticoid exposure in rats: relevance to depression. Prog Neuropsychopharmacol Biol Psychiatry.

[43] Sutton MA, Wall NR, Aakalu GN, Schuman EM (2004). Regulation of dendritic protein synthesis by miniature synaptic events. Science.

[44] Tokarski K, Urban-Ciecko J, Kossut M, Hess G (2007). Sensory learning-induced enhancement of inhibitory synaptic transmission in the barrel cortex of the mouse. Eur J Neurosci.

[45] Tokarski K, Bobula B, Wabno J, Hess G (2008). Repeated administration of imipramine attenuates glutamatergic transmission in rat frontal cortex. Neuroscience.

[46] van Gemert NG, van Riel E, Meijer OC, Fehr S, Schachner M, Joëls M (2006). No effect of prolonged corticosterone over-exposure on NCAM, SGK1, and RGS4 mRNA expression in rat hippocampus. Brain Res.

[47] Venzala E, García-García AL, Elizalde N, Tordera RM (2013). Social vs. environmental stress models of depression from a behavioural and neurochemical approach. Eur Neuropsychopharmacol.

[48] Wabno J, Hess G (2013). Repeated administration of imipramine modifies GABAergic transmission in rat frontal cortex. J Neural Transm.

[49] Whishaw IQ (2000). Loss of the innate cortical engram for action patterns used in skilled reaching and the development of behavioral compensation following motor cortex lesions in the rat. Neuropharmacology.

[50] Yuen EY, Liu W, Karatsoreos IN, Ren Y, Feng J, McEwen BS, Yan Z (2011). Mechanisms for acute stress-induced enhancement of glutamatergic transmission and working memory. Mol Psychiatry.

[51] Yuen EY, Wei J, Liu W, Zhong P, Li X, Yan Z (2012). Repeated stress causes cognitive impairment by suppressing glutamate receptor expression and function in prefrontal cortex. Neuron.

[52] Zahorodna A, Hess G (2006). Imipramine and citalopram reverse corticosterone-induced alterations in the effects of the activation of 5-HT_1A_ and 5-HT_2_ receptors in rat frontal cortex. J Physiol Pharmacol.

